# Why the Sequential Organ Failure Assessment score needs updating?

**DOI:** 10.62675/2965-2774.20240296-en

**Published:** 2024-03-25

**Authors:** Rui Moreno, Mervyn Singer, Andrew Rhodes

**Affiliations:** 1 Hospital de São José Unidade Local de Saúde São José Lisboa Portugal Hospital de São José, Unidade Local de Saúde São José - Lisboa, Portugal.; 2 Centro Clínico Académico de Lisboa Lisboa Portugal Centro Clínico Académico de Lisboa - Lisboa, Portugal.; 3 Universidade da Beira Interior Faculdade de Ciências da Saúde Covilhã Portugal Faculdade de Ciências da Saúde, Universidade da Beira Interior - Covilhã, Portugal.; 4 University College London Bloomsbury Institute of Intensive Care Medicine Division of Medicine London UK Bloomsbury Institute of Intensive Care Medicine, Division of Medicine, University College London - London, UK.; 5 St. George's University Hospitals NHS Foundation Trust & St. George's University of London London UK Adult Critical Care, St. George's University Hospitals NHS Foundation Trust & St. George's University of London - London, UK.

The Sequential Organ Failure Assessment (SOFA) score was developed almost 30 years ago. It rapidly became one of the most widely used scoring systems in intensive care, both for clinical practice and research,^(1,2)^ and remains one of the most cited scores in our speciality. Since its original description, there have been substantial changes in clinical practice that SOFA now needs to address. Clinical management has changed, and new drugs and devices have been introduced. There is a greater emphasis on noninvasive support, such as noninvasive ventilation and high-flow nasal oxygen; a wider choice of cardiovascular drugs; and greater use of extracorporeal techniques, such as renal replacement therapy and extracorporeal membrane oxygenation (ECMO). For those reasons, we decided to update the score and make it more suitable for use with current management strategies, new drugs and new devices.^(3)^

The score was designed to be easy to use and had to fulfill several guiding principles:^(1)^

1. It should be a descriptor of individual organ dysfunction during the ICU stay and not a predictor of vital status at hospital discharge for heterogeneous groups of patients for which other scores exist (e.g., Acute Physiology and Chronic Health Evaluation [APACHE], Simplified Acute Physiology Score [SAPS]). Scores for each organ system are not intended to reflect comparable mortality risk in other systems.
2. Recognizing that organ dysfunction/failure is a process rather than an event and should not be seen simply regarded as "present" or "absent" but rather viewed as a continuum; the score should characterize worsening organ dysfunction.
3. The total score should include subscores for the different organs to allow evaluation of each individual organ.
4. Variables should be rapidly and routinely obtained at every institution.
5. The number of variables should be kept low, making computation as simple as possible and encouraging daily scoring to monitor trajectories and describe the time course of the patient's illness.

For this update of the SOFA, the above principles will be retained, and a further set of principles will be added:

1. The methodology employed to update the SOFA score should reflect state-of-the-art practices, including the use of Delphi process, systematic reviews and external validation against multiple registries and databases from developed and low-middle-income countries.
2. Consideration will be given to the addition of two new organs/systems to the score: the immune and gastrointestinal systems.
3. The chosen variables should apply to every country, regardless of variations in the availability and use of drugs and devices. Each working group should include at least one member from a low-middle-income country.
4. The score should be designed to be readily computed from both electronic health records and paper records.

Some variables in the original SOFA score may be retained if they still represent the most readily available and reliable indicator(s) of function for that organ/system. On the other hand, others will need substantial revision to reflect current management practices.

Sixty intensivists from all over the world are currently involved in the development of the SOFA-2 score, including those with particular expertise in methodology, systematic reviews and database analyses. We intend to launch a preliminary version in March 2024 and a final version in autumn 2024. Therefore, we strongly encourage prospective studies that include further external validation and the incorporation of SOFA-2 into other epidemiological and research activities.
