# Supplementary material for: Why the Sequential Organ Failure Assessment score needs updating?
Source: Crit Care Sci. 2024 Mar 25;36:e20240296en. doi: 10.62675/2965-2774.20240296-en (PMC11098070; doi:10.62675/2965-2774.20240296-en)
Supplement: Translation

# Translation: Por que o escore *Sequential Organ Failure Assessment* precisa ser atualizado?

**DOI:** 10.62675/2965-2774.20240296-pt

**Authors:** Rui Moreno, Mervyn Singer, Andrew Rhodes

**Affiliations:** 1 Lisboa Portugal Hospital de São José, Unidade Local de Saúde São José - Lisboa, Portugal.; 2 Lisboa Portugal Centro Clínico Académico de Lisboa - Lisboa, Portugal.; 3 Covilhã Portugal Faculdade de Ciências da Saúde, Universidade da Beira Interior - Covilhã, Portugal.; 4 Londres Reino Unido Bloomsbury Institute of Intensive Care Medicine, Division of Medicine, University College London - Londres, Reino Unido.; 5 Londres Reino Unido Adult Critical Care, St. George's University Hospitals NHS Foundation Trust & St. George's University of London - Londres, Reino Unido.

O escore *Sequential Organ Failure Assessment* (SOFA) foi desenvolvido há quase 30 anos. Rapidamente, tornou-se um dos sistemas de pontuação mais utilizados em terapia intensiva, tanto para a prática clínica quanto para a pesquisa,^(1,2)^ e continua sendo uma das pontuações mais citadas em nossa especialidade. Desde sua descrição original, houve mudanças substanciais na prática clínica que atualmente o SOFA precisa abordar. O manejo clínico mudou e foram introduzidos novos medicamentos e dispositivos. Há uma ênfase maior no suporte não invasivo, como a ventilação não invasiva e o oxigênio nasal de alto fluxo; uma escolha mais ampla de medicamentos cardiovasculares; e mais uso de técnicas extracorpóreas, como a terapia de substituição renal e a oxigenação por membrana extracorpórea (ECMO). Por esses motivos, decidimos atualizar a pontuação e torná-la mais adequada para uso com estratégias de manejo atuais, novos medicamentos e dispositivos.^(3)^

A pontuação foi desenvolvida para ser fácil de usar e precisava atender a vários princípios orientadores:^(1)^

1. Deve ser um descritor de disfunção de órgãos individuais e não um preditor do estado vital na alta hospitalar para grupos heterogêneos de pacientes para os quais existem outras pontuações (por exemplo, *Acute Physiology and Chronic Health Evaluation* [APACHE] e *Simplified Acute Physiology Score* [SAPS]). As pontuações de cada sistema de órgãos não têm a intenção de refletir o risco de mortalidade comparável em outros sistemas.
2. Reconhecer que a disfunção/insuficiência orgânica é um processo, não um evento, não devendo ser considerada simplesmente "presente" ou "ausente", mas um contínuo; a pontuação deve caracterizar a piora da disfunção orgânica.
3. A pontuação total deve incluir subescores de diferentes órgãos para permitir a avaliação de cada órgão individualmente.
4. As variáveis devem ser obtidas de forma rápida e rotineira em todas as instituições.
5. O número de variáveis deve ser mantido baixo, tornando o cálculo o mais simples possível e incentivando a pontuação diária para monitorar trajetórias e descrever o curso temporal da doença no paciente.

Para essa atualização do escore SOFA, os princípios acima serão mantidos, e será acrescentado um novo conjunto de princípios:

1. A metodologia empregada para atualizar a pontuação SOFA deve refletir as práticas mais modernas, incluindo o processo Delphi, revisões sistemáticas e validação externa em relação a vários registros e bancos de dados de países desenvolvidos e de baixa e média renda.
2. Será considerada a inclusão de dois novos órgãos/sistemas à pontuação: os sistemas imunitário e digestório.
3. As variáveis escolhidas devem se aplicar a todos os países, independentemente das variações na disponibilidade e no uso de medicamentos e dispositivos. Cada grupo de trabalho deve incluir pelo menos um membro de um país de baixa e média renda.
4. A pontuação deve ser desenvolvida para ser prontamente calculada com base em prontuários eletrônicos de saúde e prontuários impressos.

Algumas variáveis da pontuação SOFA original podem ser mantidas se ainda representarem os indicadores de função mais prontamente disponíveis e confiáveis daquele órgão/sistema. Por outro lado, outras exigirão uma revisão significativa para refletir as práticas atuais de manejo.

Atualmente, 60 intensivistas em todo o mundo estão envolvidos no desenvolvimento da pontuação SOFA-2, incluindo aqueles com conhecimento especializado em metodologia, revisões sistemáticas e análises de bancos de dados. Pretendemos lançar uma versão preliminar em março de 2024 e uma versão final no outono do mesmo ano. Portanto, incentivamos fortemente estudos prospectivos que incluam mais validação externa e a inclusão do SOFA-2 em outras atividades epidemiológicas e de pesquisa.
